# Overtaxed Brains*Published in Buffalo *Courier*, July 30th, 1882. Revised for re-publication.

**Published:** 1883-01

**Authors:** R. R. Gregg

**Affiliations:** Buffalo


### OVERTAXED BRAINS.*

### A PHILOSOPHICAL AS WELL AS MORAL LESSON FROM A SCIENTIFIC STANDPOINT.

R. R. Gregg, M. D., Buffalo.

The Mr. Pease referred to in this article was the noted pianist and composer of our city, Mr. Alfred H. Pease. He had always led a very exemplary life and worked excessively hard in his profession—so much so that he commonly had a half-dazed expression of countenance. He went to Chicago in May last and played a very successful engagement for a week, then went or wandered off to St. Louis, where his friends lost all trace of him for several weeks, until his dead body was found under the following circumstances: A man was seen to fall in the streets of that city one day and died soon after. An inquest was held, but no knowledge obtained as to who he was until a reporter accidentally recognized his body at the Morgue as that of Mr. Pease, from the descriptions he had read of him. Then it was learned he had been on a debauch there for weeks, and from all accounts the first in his life. He had evidently lost all moral control of himself through his excessive intellectual efforts for years and through the physiological process above pointed out. What a pity, therefore, he could not have been found and properly cared for until restored to his former self again!

In an editorial upon the fate of Mr. Pease in Sunday's *Courier* of July 16th we read:—"He was, indeed, almost the last man under the sunny exterior of whose nature one would have suspected a lurking mystery of evil. Let the sod cover his faults, and his fate and his memory survive only in his music." The words in this quotation,—"lurking mystery of evil," and "let the sod cover his faults," I take as the text of a discourse which I have long felt should be written by some one who could give us something true to nature, or, at least, a modicum of truth upon the subject of overtaxed brains, and which, from not a little individual experience and no one appearing to undertake the task, I feel it a duty to essay now myself, even though it may be done in a very imperfect and unsatisfactory manner.

I was not acquainted with Mr. Pease, excepting to know him by sight, and knew nothing even by hearsay of his daily habits or of occasional harmful indulgencies, if, indeed, he were guilty of such,

* Published in Buffalo *Courier*, July 30th, 1882. Revised for re-publication.

therefore, I do not feel called upon to defend his memory either on the score of acquaintance or from knowledge of facts creditable to his daily life. But I take up the subject on a much broader ground and take him as a type of a large and rapidly increasing class of mentally overworked men who, in their acts, if not otherwise, are daily calling aloud for help, and should receive it. "They know not what they do," and should be instructed in the right. In other words, they should be given a clear and simple philosophy on the subject of mental overwork by which they can guide themselves before it is too late, or by which others can guide them if they have gone too far.

From the little knowledge I did have of Mr. Pease, and simply in his capacity as a prominent musician and composer, I take it that his most harmful indulgencies (harmful to himself, I mean) were in the excessive study to perfect himself and excel in his chosen profession. Judging him by sight and by his accomplishments in his art, his mind was wholly absorbed in that direction to the exclusion of almost everything else.

This exclusive devotion to one thing or to one line of thought year after year is always very wearing, as all know; but it is also the rock on which many a devotee is *morally* wrecked, which few or none seem to know.

To illustrate: Whether phrenology be true or not, there are three great divisions of the human mind which all recognize, namely, intellectual, moral, and immoral or animal. And it may be safely assumed that each of these manifestations of mind has its special seat of activity in or depends upon some special division of the brain. Indeed, it has come to be generally regarded that the anterior or frontal brain is the seat of intellectual action, the superior or top brain the seat of the moral sentiments, and the posterior and base of the brain the home of animal forces.

Again, it is certain that the blood carries to and furnishes the brain with the nutriment upon which the various operations of the mind depend. If there were no activity of nutrition in the cells of the brain there could be no activity of thought, whether good or bad. And if no blood were carried to the brain there could be no activity in the nutrition of its cells, which is a negation of thought.

This, then, leads us to the inquiry, What part of the blood nourishes the brain? The answer is, a portion of the fatty matters and a portion of the salts. How much of these are there in the blood? Why, there are not to exceed *eight* parts of fatty matters and salts combined in *one thousand parts* of blood (less than one part of both in one hundred of blood), and yet even this little cannot all be used in the nutrition of the brain, as a portion of both must be used for other purposes. The salts must be used in part for the nutrition of the bones and also somewhat for the muscles, etc., while the fatty matters must be partly used in deposits to facilitate the movements of parts upon each other and as cushions to other parts, also as a covering for protection to organs, like the bowels against cold, etc., etc. (And here let me say, in passing, that the reason why very active intellectual people, especially those of quick, exhausting, nervous activity, are so commonly spare, or at least not corpulent, is because they use up so much of the fatty matters of the blood in the brain that not enough of them are left for deposits to make them plump, or for protection against cold, hence such are generally so easily chilled.)

Now, for the application of these facts to our moral natures: Supposing a man like Mr. Pease, any man in fact, to apply himself so excessively to one or a few lines of intellectual thought that he uses up all the brain-nourishing material of the blood in the intellectual part of the brain and leaves none of it, or a very insufficient supply, to sustain the moral part of it, what must be the consequences? Why, in that case, of course, the moral organs are starved, consequently cannot put forth any vigor of moral sentiment, which leaves the victim for the time being more or less completely without a moral guide, until nature can have time and opportunity to establish a reaction that will set things going on again in their normal course; that is, until the moral organs are allowed to receive their full proportion of food again and be set right thereby. But here is another highly important point to be considered. Nature always takes care of herself first, that is, her greatest concern and effort is to save the life of the individual first, leaving wrongs against her better work to be rectified afterward. But as life depends upon the activity of the animal forces, which have also in these cases been robbed of their support along with the moral organs, and she being unable, as things are constituted, to preserve life excepting through the animal parts of the brain, she arouses these to activity first; that is, feeds them before she does the moral organs, and it is during this interim, be it long or short, that the one who has thus so excessively overtaxed his brain intellectually is necessarily deprived of the requisite moral support within himself and the animal has sway. And then is when he should have the proper moral support from without, and from those who know, or should know, *what they are about and what to do.*

Here, then, is the "lurking mystery of evil" that may be developed in almost any of us in that way, the "fault" that the sod, or the mantle of charity, should cover so deeply in such cases that not the slightest allusion should ever be made to it afterward in censure, in ridicule, or in jest, and not even in earnestness, unless it were to raise the note of warning in time to prevent the transgressor, if he still lived, from a repetition of the causes of his misfortunes in subsequent long-continued, exhausting mental work.

The writer has never had this part of the experience, as he knew beforehand what must come of it and shunned it; but he has seen times that he would almost have given worlds, if possessed of them, for relief.

As bad or worse off still is the victim that falls into the hands of a physician who knows little or nothing of these things, and does not appreciate the great efforts nature is making through sleeplessness, depression, melancholy, and other ways to save his patient, but drugs him with morphine, chloral, bromides, stimulants, etc., as the case may be, until all her efforts are paralyzed and she loses the mastery. The writer was once told by a medical superintendent of an insane asylum that during the years he had charge thereof in not one instance where the patient had been freely drugged with the agents named before his admission was he cured, whereas those not so drugged were quite a large proportion of them cured. Softening of the brain, paralysis, apoplexy, or other physical disease and wreck are often also as legitimate results of such drugging as are imbecility, suicide, or incurable insanity, all of which are commonly produced by it.

When will physicians see and learn that the natural road back to a healthy and vigorous moral activity of those who are naturally moral, but have lost control in the ways named, is through depression, melancholy, and other more or less similar means? "Suffer and be strong," through nature's vigorous reactions, is certainly the motto here unless the suffering is carried too far and unless nature is stopped in her beneficent work by drugs. And here let me tell the sufferer that his clearest and best thoughts will come after such suffering and reaction if he has not given cause for the former to be too great, and he be not drugged. Drugging, however, will destroy subsequent clear thoughts for days, if not weeks, and make the ultimate suffering much greater than it otherwise would or could be. If the suffering becomes too great there are other ways of relieving it than by drugging.

If any should doubt that through irritability, extreme nervous sensitiveness, melancholy, etc., lies the royal road back to the normal conditions in many cases, let me cite them the almost universal fretfulness, crossness, and irritability of children when over-tired from play, or when recovering from sickness, as the proof. The universality of this process of nature in restoring children to normal conditions proves what it means, and shows us of what great value a proper application of the fact to the guidance of the mentally overworked might be. The child could not be restored to health and vigorous activity when seriously overcome by excessive efforts or disease without going through the reactive efforts named; the mentally overworked cannot get back to their proper moral basis without melancholy and other efforts at reaction; then how wrong to stimulate, drug, or otherwise destroy this, the only process of restoration!

Still further, if any doubt that sleeplessness is one means of restoration to a greatly overworked brain, let me tell them of a little individual experience. Often has the writer lain awake at night, hours upon hours together, with thoughts running at race-horse speed in consequence of the blood being drawn in such great excess to the thinking organs to sustain a prolonged intellectual effort, until finally exhaustion would follow or the excitement exhaust itself; then an aching of the limbs arose that would tell of the coming reaction; then profound sleep, a few hours of which would restore the exhausted mental energies and vigor of thought the same as before. And there was where a halt for a day or two ought to have been called each time to have saved much more severe subsequent suffering, but was not. Supposing, on the contrary, an anodyne had been taken to force sleep and thus stopped the natural process of reaction, this would not have come in days, if in weeks, and the sufferings to get out from under the effects of the anodyne would have been much greater than the lying awake, while under a frequent repetition of this violation of nature the writer would not have been here to have told his story, or would have been rendered incapable thereby of telling it.

In conclusion, the facts, or claimed facts, on moral issues given in this discourse must not be held to excuse the naturally depraved in their wrong-doing or to tolerate their continuance in depravity; but to those who have exhausted themselves in *well-doing* until immoral acts (but generally to themselves) have resulted, such facts afford a rational basis for excuse until the transgressor can be restored to his proper and natural moral basis. And it is hoped that this discussion of the subject may enlighten all more or less in the matter and help not a few who are now suffering, or those who may suffer in the future, to a clearer understanding of their cases, and thereby aid them in getting back to their former selves again.

The "fault" therefore, if fault there be, is in the original transgression, or in violation of the harmony which nature has set up in all moral minds, by such excessive application to intellectual labor that the moral part of the brain is robbed of the sustenance required to enable it to act with vigor and hold control; hence the temporary lapse to purely animal indulgences, as shown, until the wrong is righted. But the victim knows nothing of a moral wrong being involved in that original step. Indeed, who does? The ignorance that prevails upon the subject in this light is something worse than "Egyptian darkness." The books upon mental and moral philosophy, upon psychology and insanity, which have fallen under the writer's observation are as innocent of any such teaching upon their respective themes as unborn babes are of such conceptions. Authors and readers alike are equally ignorant, yet who shall say that the simple facts here presented should not be made at once the corner-stone of a new philosophy upon the subject, by which all may be guided and through which all may be saved?

Who now thinks that any moral wrong, or even harm, can come, excepting to the individual's physical health, from excessive mental application to any of the great elevating walks of life? All think that the more excessive the application the more commendable the efforts so long as the physical health holds out, little knowing that the worst results, or even actual crimes, may come of it, if, indeed, we may call the resulting acts crimes.

But nature is ever kind, or ever tries to be, in avoiding or trying to avoid by various devices such immoral extremes being reached, except it be after years of great transgressions, and even then, if left to herself or not drugged, she often succeeds in her purposes against the wrong. For instance, she sets up all kinds of counter-actions to tide over the emergency. She permits of the exhaustion of the intellectual forces and thus stops the transgressor by rendering him unable to think or study until time has been given for a recuperation to come that will restore the exhausted brain to its normal condition before the moral forces have been reached to be seriously harmed. She forces sleepiness and gets recuperation through sleep in the early stages of such cases. Or, when the violator has pushed his violations too far and overcome that obstacle to his work, then she sets up sleeplessness to further exhaust and stop him in that way. She disgusts him with the subject and thus gets time for repairs. She often excites severe headaches to stop the excessive work. She establishes great irritability of mind to thereby divert him from his purposes, and here the animal steps in to try and right things. She sometimes arouses great fear through unnatural and strange sensations and stops him in that way. She quite commonly sets up physical diseases as a consequence of the mental efforts depriving the blood too rapidly of its fatty matters and salts, and thereby destroying its normal constitution, that is, the proper proportion among its constituents, until physical disease or physical pain is produced to divert his attention. This applies very commonly to the earlier violations in this direction, as in school children who are urged on to excessive study to their great injury (which it is hoped both teachers and parents will deeply consider), although it is a frequent result to older transgressors as well. And last, but not least, she very commonly and more or less fully destroys hope for the time being, through that part of the brain which is the seat of our hopeful natures being starved so that it cannot act, and thus produces the "blues," or melancholy, to thereby stop the too close and excessive mental application.

But for these various devices being set up for the purpose stated, the moral and physical wrecks of men from mental overwork would be much more numerous than they are now, although now far too numerous and rapidly increasing. Indeed, but for such guards over us, many men would be destroyed where one is now, and few or none who become so wholly absorbed in any intellectual pursuit would be safe from moral degradation, from suicide, from insanity, or from premature death through disease. Having had not a little personal experience, as well as some professional observation, in many of these efforts of nature to avert worse results, the writer feels that he knows something of what he speaks and has not overdrawn the picture.

Such, then, is nature's intelligent and practical way of dealing with this subject. Let us now contrast with it man's ignorant and stupid way of trying to cheat nature out of her reasonable and just demands and stop her restorative efforts. Among the most common devices of man to right this wrong against nature is the resort to alcoholic stimulation. But, alas for the victims who get started in this direction! Three-fourths or more of them are sure of moral wreck. In addition to the mastery that alcohol is liable to get through the appetite over every one who indulges therein, it stimulates the intellectual faculties to greater work for the time being than they could otherwise perform, thereby using up more of the fatty matters and salts of the blood in the intellectual organs and starving the moral organs more than nature would otherwise allow. Matters then go on from bad to worse, more work and more drink "to keep upon," until the moral sentiments are lost through simple want of food to sustain them, and the subject has lost all self-control and becomes a sot in spite of anything he can do to avert it. And here nature again tries to save herself and the victim through the inactivity and stupidity that excessive drinking brings to cut off all mental action, but the appetite has become so strong while under the loss of moral control that it is then often impossible, or nearly so, to subdue it.

